# Exploring the N-Glycosylation Profile of Glycoprotein B from Human Cytomegalovirus Expressed in CHO and *Nicotiana tabacum* BY-2 Cells

**DOI:** 10.3390/ijms20153741

**Published:** 2019-07-31

**Authors:** Nicolas Smargiasso, Joseph Nader, Stéphane Rioux, Gabriel Mazzucchelli, Marc Boutry, Edwin De Pauw, François Chaumont, Catherine Navarre

**Affiliations:** 1 Mass Spectrometry Laboratory-MolSys, GIGA Proteomics Facility, University of Liège, 4000 Liège, Belgium; 2 Louvain Institute for Biomolecular Science and Technology, UCLouvain, 1348 Louvain-la-Neuve, Belgium; 3 GlaxoSmithKline Vaccines, 1330 Rixensart, Belgium

**Keywords:** glycoprotein B, cytomegalovirus, plant cell suspension culture, N-glycosylation, mass spectrometry

## Abstract

The ability to control the glycosylation pattern of recombinant viral glycoproteins represents a major prerequisite before their use as vaccines. The aim of this study consisted of expressing the large soluble ectodomain of glycoprotein B (gB) from Human Cytomegalovirus (HMCV) in *Nicotiana tabacum* Bright Yellow-2 (BY-2) suspension cells and of comparing its glycosylation profile with that of gB produced in Chinese hamster ovary (CHO) cells. gB was secreted in the BY-2 culture medium at a concentration of 20 mg/L and directly purified by ammonium sulfate precipitation and size exclusion chromatography. We then measured the relative abundance of N-glycans present on 15 (BY-2) and 17 (CHO) out of the 18 N-sites by multienzymatic proteolysis and mass spectrometry. The glycosylation profile differed at each N-site, some sites being occupied exclusively by oligomannosidic type N-glycans and others by complex N-glycans processed in some cases with additional Lewis A structures (BY-2) or with beta-1,4-galactose and sialic acid (CHO). The profiles were strikingly comparable between BY-2- and CHO-produced gB. These results suggest a similar gB conformation when glycoproteins are expressed in plant cells as site accessibility influences the glycosylation profile at each site. These data thus strengthen the BY-2 suspension cultures as an alternative expression system.

## 1. Introduction

The development of vaccines against enveloped viruses is often based on recombinant antigens corresponding to viral glycoproteins spiking on their surface. Glycans on viral envelope glycoproteins play important roles in virus biology. Results obtained from N-glycoprofiling of various viral proteins as well as glycoproteomic studies showed that most of the putative N-glycosylation sites are glycosylated with high occupancy, and that the host cell line used to produce the viral glycoprotein might have a strong influence on the glycosylation profile [1]. A number of viral glycoprotein vaccine candidates have been successfully expressed in plants either transiently in *Nicotiana benthamiana* leaves or stably in transgenic plants, and are being evaluated in clinical trials [2]. For instance, Medicago Inc. has developed a transient plant expression platform for the production of influenza virus hemagglutinin (HA) virus-like particles that are currently in phase 3 [3].

Plant suspension cells, such as *Nicotiana tabacum* Bright Yellow-2 (BY-2) cells, constitute an alternative technology for producing recombinant therapeutic N-glycosylated proteins [4,5]. The first glycoprotein derived from plants that has been approved for human use (Elelyso, a recombinant human glucocerebrosidase) is produced in carrot suspension cells [6]. Several other recombinant glycoproteins produced in BY-2 cells have reached clinical trials [7]. However, only very few viral glycoproteins have been produced in this platform so far, like the Hepatitis B surface Antigen in soybean and tobacco NT1 cell suspension cultures [8].

When the glycoprotein of interest is secreted into the culture medium, the purification process can be simplified and high consistency of the N-glycan profile is expected since the glycoprotein should have been thoroughly processed into complex N-glycans in the Golgi before reaching the culture medium. Expression of the house dust mite Derp1 allergen in tobacco BY-2 cells led to the accumulation of a proteolytically maturated and correctly folded protein in the culture medium with the N-glycosylated Asn150 decorated with both oligomannose-type N-glycans and plant complex N-glycans [9]. The glycosylation profile of the human secreted alkaline phosphatase (one N-site) produced in tobacco NT1 suspension cells indicated the presence of GnGnXF, GnMXF and MMXF (for nomenclature, see Figure S1), altogether representing about 85% [10]. Analysis of the glycosylation profile of three different IgG antibodies secreted in the extracellular medium of BY-2 cells confirmed a relatively homogenous N-glycosylation profile with a high proportion (between 67 and 87%) of the complex glycoform GnGnXF decorating the conserved Asn297 [11,12,13]. Similarly, N-glycans released with Peptide-N-Glycosidase PNGase A from recombinant human DNaseI (two N-sites) secreted in the BY-2 culture medium correspond mainly to GnGnXF (51%) and GnMXF (45%) [14]. Similar relative proportions were reported for N-glycans released from human cytotoxic T-lymphocyte antigen 4-immunoglobulin (hCTLA4Ig; three N-sites) secreted in the culture medium of rice suspension cells: GnGnXF (49%) and GnMXF (44%) [15]. Analysis of N-glycans released from human acid alpha-glucosidase secreted from rice suspension cells also showed the presence of a large amount of complex bi-antennary structures like GnGnXF (49%) and GnGnX (12%), some paucimannosidic structures (e.g., GnMX (14%), GnMXF (8%)), as well as a small proportion of Lewis A-containing structures (e.g., Le^a^GnXF (7.5%)) [16]. This glycoprotein contains seven putative N-sites, and there is no information on the differences between the seven sites or on the microheterogeneity of each specific glycosite. All these glycoprofile data concerning secreted recombinant glycoproteins are compatible with the N-glycan processing steps generally accepted for plants [17].

The case of recombinant proteins with several glycosylation sites and specific heterogeneity at each site raises an interesting question: is there any similarity in this site-specific heterogeneity between plant and animal-produced proteins? For instance, is an N-site that is poorly processed (e.g., with high mannose content) in animal cells also poorly processed in plant cells? This kind of analysis has already been addressed for some glycoproteins of virus envelope produced in plant leaves, like influenza hemagglutinin virus-like-particles H1-VLP and H5-VLP, which display complex structures like GnGnXF or Le^a^Le^a^XF on each of the six N-glycosylation sites as studied by site-specific glycoproteomics [18]. Another example is the heavily glycosylated HIV gp140, which contains a high proportion of oligomannosidic structures (80%) and was reported to react with 2G12, an antibody specifically targeting a glycan-dependent epitope on the outer domain of the glycoprotein [19]. The envelope glycoprotein B (gB) of the human cytomegalovirus (HCMV) is another interesting model because it contains 17 or 18 N-glycosylation sites (depending on the strains [20]) and shares structural and functional properties with gB proteins from other herpesvirus [21]. In addition, this protein is the most extensively studied recombinant vaccine against HCMV [22,23]. gB is a type I glycoprotein consisting of four distinct regions: a large ectodomain (25–697), a membrane proximal region (698–750), a transmembrane domain (751–773) and a cytoplasmic domain (774-906). Crystal structures of the gB ectodomain have recently been resolved and revealed a spike-like trimer [20,24]. HCMV subunit vaccines incorporating soluble variants of gB (truncated at the transmembrane domain and having a targeted deletion of the furin proteolytic site) have been under development for several years [22,23,25]. As such, the vaccine formats (derived from the Towne, Merlin or AD169 HCMV strain sequences) were expressed as a secreted single polypeptide in Chinese hamster ovary (CHO) cells, and the protein was purified by chromatography from the culture supernatant [23,25]. As pointed out above, an outstanding feature of the gB ectodomain resides in its complex glycosylation pattern since this protein is extensively glycosylated with seventeen predicted NXS/T-glycosylation sites conserved across 60 HCMV clinical and laboratory-adapted strains [20]. Crystal structure indicated that within the ectodomain from the AD169 strain expressed in insect Sf9 cells [20] or the ectodomain from the Merlin strain expressed in Human Embryonic Kidney HEK293GnTI^−^ cells [24], all of the N-sites within the resolved regions of the polypeptide contain glycans in the structure. Previous studies using inhibitors of glycosylation and endoglycosidases in HCMV(AD169)-infected Human fibroblasts indicated that the fully processed gB protein contains both oligomannosidic and complex N-glycans [26]. However, to the best of our knowledge, no N-glycoproteomics of any gB protein has been reported so far. On the other hand, O-glycoproteomic analysis of HCMV(Towne)-infected fibroblasts identified two O-glycosites at the N-terminus of the gB protein (T58 and S64) [27].

The full-length HCMV gB protein of the Towne strain had already been successfully expressed as a membrane protein in the protein storage vesicles of tobacco and rice seeds, representing about 1% of total seed protein, but no biochemical characterization was reported [28,29,30,31]. Here, we produced the gB ectodomain from the AD169 strain in *N. tabacum* BY-2 transgenic cell lines. Recombinant gB accumulating in the culture medium was semi-purified and subjected to a comprehensive, site-specific, quantitative N-glycosylation analysis. In parallel, we also determined the N-glycosylation profile of the same gB polypeptide secreted from CHO cells. This comparative analysis reveals that, apart from their respective specific residues, there is a striking parallelism of the glycoprofile between the CHO- and BY-2-produced gB.

## 2. Results

### 2.1. Expression of HCMV gB Ectodomain in Nicotiana tabacum BY-2 Cells

**Figure 1:**
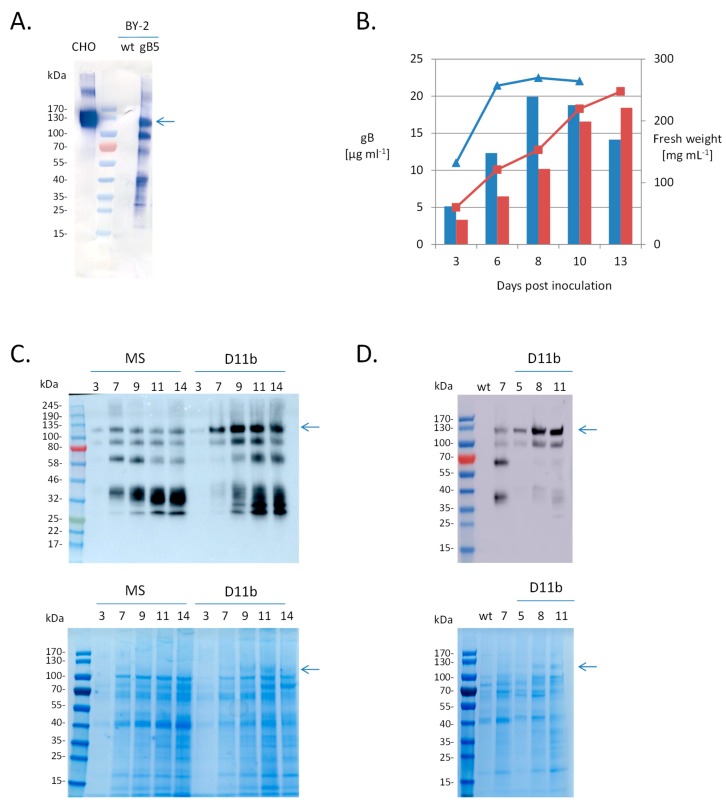
Expression and secretion level of glycoprotein B (gB) ectodomain in Bright Yellow-2 (BY-2) cells. (**A**) Western blotting analysis of gB. CHO, purified gB produced in Chinese hamster ovary (CHO) cells (1.2 µg); wt, extracellular medium from BY-2 wild type (40 µL); gB5, extracellular medium from line gB5 (40 µL). Western blotting using anti-gB antibodies was performed as indicated in Materials and Methods. (**B**) Kinetics of cell growth (fresh weight-lines) and gB accumulation in culture medium (quantified by ELISA-bars) of line gB5 grown in MS (blue) or D11b medium (red). (**C**) Analysis of extracellular medium of line gB5 grown in MS or D11b medium by Western Blotting (10 µL) and Coomassie-blue stained SDS-PAGE (40 µL). Samples of extracellular medium from line gB5 were collected 3, 7, 9, 11 and 14 days after inoculation. Western blotting using anti-gB antibodies was performed as indicated in Materials and Methods. (**D**) Analysis of extracellular medium of line gB16 grown in MS or D11b medium by Western Blotting (50 µL) and Coomassie-blue stained SDS-PAGE (50 µL). Samples of extracellular medium were collected 7 (MS), or 5, 8 and 11 (D11b) days after inoculation. Western blotting using anti-gB antibodies was performed as indicated in Materials and Methods.

*N. tabacum* BY-2 suspension cell lines that constitutively express the gB soluble region (gB64-724) containing most of the ectodomain were screened by analyzing the culture medium by Western blotting and ELISA to identify the best-producing line. BY-2-produced gB displayed a band with an apparent size of 130 kDa, similar to the band observed for the CHO-produced gB control (Figure 1A). Taking into account the predicted size of the polypeptide (72 kDa), this suggests that N-glycans account for about 45% of this mass, which is compatible with the large number (18) of predicted N-glycosylation sites in the ectodomain of AD169 strain. For the BY-2-produced gB, additional bands of a smaller size were also detected, possibly resulting from proteolytic cleavage. Line 5 was chosen for further characterization.

Accumulation of gB in the culture medium was compared for the gb5 line cultivated in either the conventional Murashige and Skoog MS medium or the D11b medium, which was optimized for the accumulation of a human antibody in BY-2 cells [32]. BY-2 growth was delayed in D11b medium and the stationary phase was reached only 13 days (compared to 7 days in MS medium) after subculture, but the total amount of gB quantified by ELISA gave a similar value in the two mediums (20 mg/L (MS 8 dpi) and 18 mg/L (D11b 13 dpi)) (Figure 1B). However, qualitative analysis by Western blotting showed that the 130 kDa gB band was more abundant in the D11b medium (Figure 1C). As a consequence, this band was visible in the SDS-PAGE gel stained with Coomassie blue. This was also the case for two other gB lines tested (Figure 1D).

### 2.2. Purification of BY-2-Produced gB

**Figure 2:**
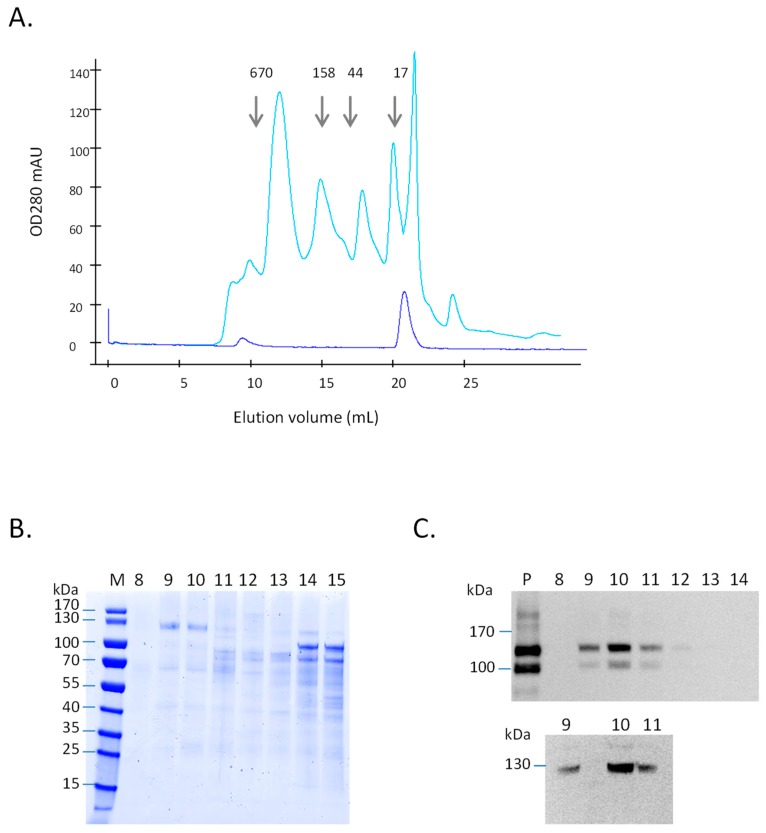
Purification of gB ectodomain produced in BY-2 extracellular medium by SEC. (**A**) Overlay of size exclusion chromatograms of line gB5 extracellular medium (corresponding to 20 mL precipitated with (NH_4_)_2_SO_4_—light blue) and purified CHO-produced gB ectodomain (7 µg—dark blue). The elution volumes of the 670 kDa, 158 kDa, 44 kDa and 17 kDa standards are marked with arrows. (**B**) Coomassie-blue stained SDS-PAGE of fractions eluted from SEC (Size-Exclusion Chromatography). M: Precision Protein Color Standards. Fractions 8–15 eluted from size exclusion chromatography (40 µL) of extracellular medium line gB5. (**C**) Western blot analysis of fractions eluted from SEC. Top, P: line gB5 extracellular medium precipitated pellet (10 µL—corresponding to 0.7 mL of extracellular medium); Fractions 8–12 collected after SEC of extracellular medium from gB line 5 (40 µL). Bottom, Fractions 9–11 collected after SEC of CHO-produced gB (40 µL). Western blotting using anti-gB antibodies was performed as indicated in Materials and Methods.

In spite of its C-terminal 6xHis-tag, BY-2-produced gB could not be purified using metal affinity chromatography (data not shown). This behavior had already been reported for other HCMV proteins expressed in insect cells [21,33] and was interpreted as a poor accessibility of the tag in the folded protein. We therefore proceeded via precipitation of the extracellular proteins with ammonium sulfate followed by size exclusion chromatography (Figure 2A). Analysis of the fractions by SDS-PAGE (Figure 2B) and Western blotting (Figure 2C) revealed that BY-2-produced gB was eluted as a peak near the void volume, in the vicinity of the 670kDa marker. Interestingly, the purified CHO-produced gB eluted in the same fraction when applied on the same size exclusion column (Figure 2A–C).

### 2.3. N-Glycan Repertoire of CHO-Produced gB

**Figure 3:**
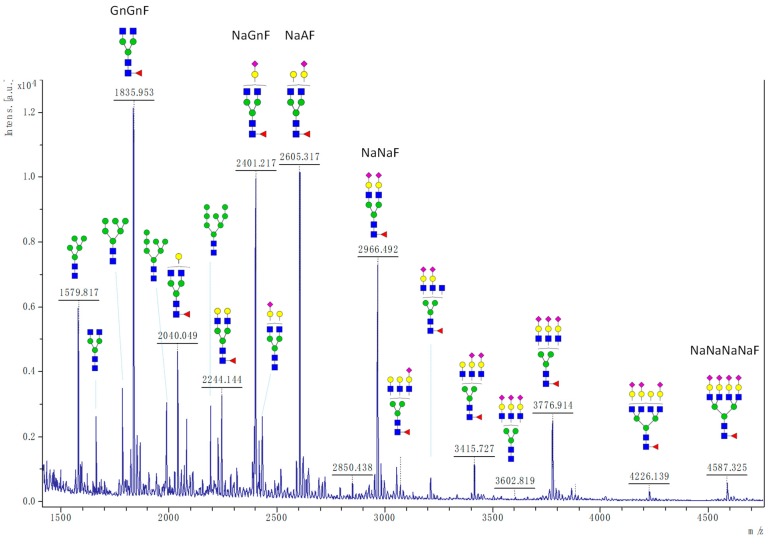
Global N-glycan repertoire of gB ectodomain produced in CHO cells. Glycans are annotated with cartoons above main peaks as recommended [34].

Because little is known about the glycan moiety of gB, we first assessed the N-glycosylation status of gB64-724 produced in CHO cells. Digestion using PNGaseF was used to release N-glycans from purified gB. MALDI-TOF mass spectrometry analysis of the permethylated N-glycan pool showed that the global N-glycan repertoire was particularly extended with more than 60 peaks corresponding to N-glycans (Figure 3 and Table S1). The most intense ones were attributed to the complex-type bi-antennary glycoforms GnGnF (*m*/*z* 1835.953: 11.8%), NaGnF (*m*/*z* 2401.217: 9.6%), NaAF (*m*/*z* 2605.317: 10.1%), and NaNaF (*m*/*z* 2966.492: 8.0%). Some complex tri- and tetra-antennary structures were also detected including heavily sialylated N-glycans like the structure NaNaNaNaF (*m*/*z* 4587.325: 1.5%). On the other hand, several peaks corresponding to oligomannosidic N-glycans were identified. This suggests that N-glycans decorating the 18 putative gB N-sites were probably differentially processed in the Golgi according to their localization in the protein and their accessibility to the glycosyltransferases and glycosidases.

### 2.4. Site-Specific N-Glycan Analysis

In order to investigate the N-glycosylation profile at each glycosite, we then performed N-glycoproteomic analysis of gB produced in both CHO and BY-2 cells by proteolysis followed by LC-MS/MS analysis. The gB64-724 ectodomain contains eighteen predicted N-glycosylation sites (NXS/T): N68, N73, N85, N208, N281, N286, N302, N341, N383, N405, N409, N417, N447, N452, N464, N465 (not conserved in all HCMV strains), N554, N585. Using trypsin proteolysis, glycopeptides corresponding to nine putative N-sites (N85, N281, N286, N302, N341, N383, N405, N409, N554) were identified thanks to the presence of cleavage sites located close to the processed N-site. In order to increase the number of N-sites analyzed, we used a recently developed MultiEnzymatic Limited Digestion protocol [35], which allowed the detection of glycopeptides corresponding to eight additional N-sites (N68, N73, N208, N417, N452, N464, N465, N585). No glycopeptides matching site N447 were detected, regardless of the digestion protocol used, but this does not necessarily indicate that this site is not glycosylated. An unexpected cleavage at residue Q458 (located in the mutated furin cleavage site) was observed, allowing us to detect glycopeptides that contained site N452 in both samples (CHO and BY-2) and glycopeptides that contained sites N464 and N465 in the sample derived from CHO. The occurrence of this unexpected cleavage site was first identified in deglycosylated samples using a “no enzyme” database search (thus considering every amino acid as a potential cleavage site). The glycopeptides data set was then re-examined for these glycopeptides. Mass spectra that confirmed their presence are presented in supplementary data (Figure S2). Finally, due to the close proximity of some N-sites, two sets of glycopeptides contained two predicted N-sites (N405-N409 and N464-N465). As a result, the glycoforms identified on those peptides could not be assigned to any individual N-site.

**Figure 4:**
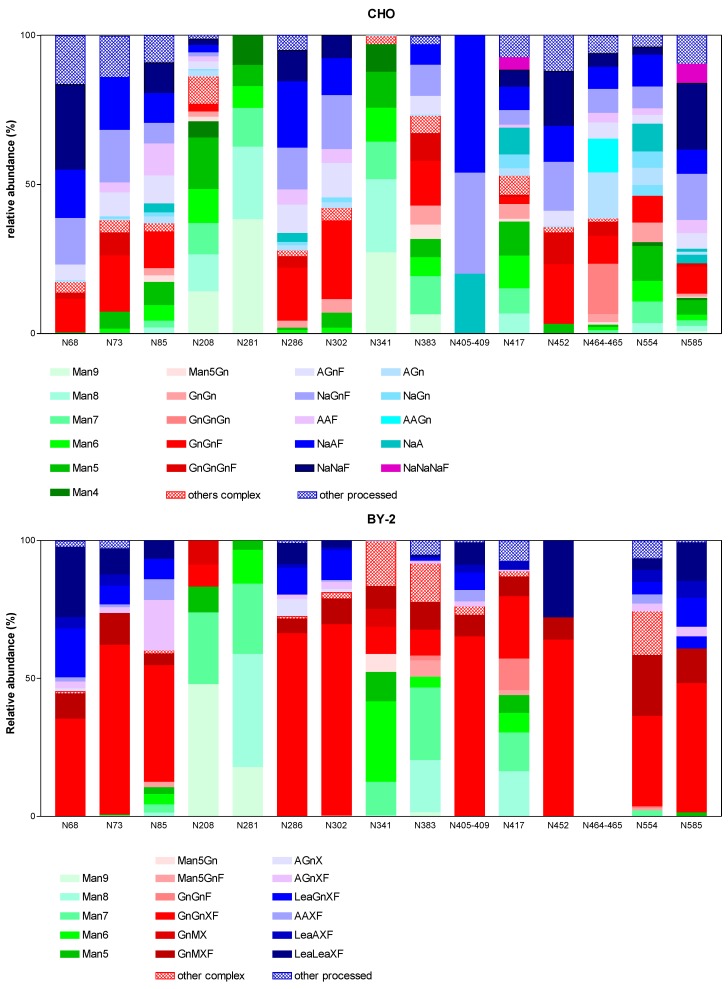
Quantification of the relative abundance of N-glycans detected by mass spectrometry at each glycosite on gB ectodomain expressed in CHO and BY-2 cells. N-glycans detected (>5%) are represented and abbreviated according to the ProGlycAn system. The quantitative data derived from a single analysis for both samples. Glycans are assigned into three groups: (1) oligomannosidic structures (in green); (2) hybrid/complex structures exhibiting terminal GlcNAc (in red); (3) highly processed complex-N-glycans (in blue). Glycans present in less than 5% are grouped in “others” and are hatched.

For each putative N-site, the relative intensity of every N-glycopeptide ion was calculated (data in Table S2). A striking observation is the high number of different glycoforms observed at each N-site. In order to simplify the comparison of data obtained from both CHO and BY-2 cell lines, glycoforms were assigned to three main groups: (1) oligomannosidic structures, (2) hybrid/complex structures exhibiting terminal GlcNAc antennae, i.e., without additional galactose and/or sialic acid (in CHO) or Lewis A epitope (in BY-2) and (3) highly processed complex N-glycans, i.e., with additional galactose and/or sialic acid (in CHO) or Lewis A epitope (in BY-2). For CHO cells, some complex and highly processed glycans contain three or four GlcNAc, suggesting tri- and tetra-antennary structures (or bisected structures for some glycans). This is particularly the case for N68, N73, N383, N452, N464-N465 and N585 where these tri- and tetra-antennary structures account for more than 20% of all glycans. On the other hand, as anticipated, these structures are completely absent in the BY-2-derived sample and this can at least partially explain the lower number of glycans observed in BY-2 cells. Moreover, detailed studies revealed a large microheterogeneity with more than twenty distinct structures identified at specific N-sites (summarized in Figure 4). The glycosites N208 and N281 are mainly decorated with oligomannosidic structures in both CHO and BY-2 samples. This is also the case, but to a lesser extent, for N341, N383 and N417.

On the other hand, the glycosites N68, N73, N85, N286, N302, N405-N409, N452, N554, N585 were mostly decorated with hybrid, complex and paucimannosidic N-glycans in the BY-2 sample. Some Lewis A-like structures were also observed but to a smaller extent. In comparison, in CHO cells, the same glycosites were mainly decorated with highly processed structures (containing beta-1,4-galactose and sialic acid) and a relatively low proportion of hybrid and complex structures (this observation is particularly striking for the glycopeptides containing N405-N409). Taken together, these data show a globally similar N-glycan distribution over the gB proteins produced in CHO and BY-2 cells.

### 2.5. Glycosite Occupancy

The CHO- and BY-2-derived gB samples were digested with trypsin and then PNGase F (CHO) or PNGase A (BY2) was added to release all types of N-glycans. This PNGase treatment resulted in the deamidation of asparagine (N) in the NXS/T sequence of the glycopeptides and the resulting aspartic acid (D) was used as an N-glycosylation signature. The ratios of the MS signals of the resulting deamidated peptides (glycosylated) to unmodified (non-glycosylated) peptides were considered as the glycosylation efficiency (Table 1). The occupation level was found to be similar between both production platforms even for the sites showing partial occupancy. Another observation was that sites N73 and N405 exhibited occupancy of only 43% (CHO) and 54% (BY-2) for N73, and 71% (CHO) and 64% (BY-2) for N405. These two sites are very close to N68 and N409, respectively, two N-sites that displayed a high occupancy rate. This suggests that the latter are probably N-glycosylated more efficiently than the adjacent N73 and N405.

## 3. Discussion

We have produced the gB ectodomain from HCMV secreted in the culture medium of BY-2 cells. The elution profile of the protein observed on the size exclusion chromatography column was similar to that obtained for the same gB protein produced in CHO cells. Since the CHO-produced gB was not precipitated by ammonium sulfate prior to the size exclusion chromatography analysis, the elution of the gB ectodomain near the void volume cannot be attributed to a salt-induced protein aggregation [21] and [20] have noted that gB trimers associate in higher molecular weight structures, presumably through hydrophobic interaction between hydrophobic residues in the two fusion loops. Mutation of several hydrophobic residues in the fusion loop regions decreased the formation of rosette-like aggregates and resulted in a gB structure restricted to trimers [20,21].

Our study shows that most of the putative N-glycosylation sites of the gB ectodomain are glycosylated with similar high occupancy in both CHO and BY-2 cells. This demonstrates that CHO- and BY-2-expression systems are similarly efficient in attachment of N-glycans. This is remarkable since it has been shown that the site occupancy can vary in different producer cell lines [1]. Moreover, the elevated occupancy rate observed for the gB ectodomain expressed in BY-2 cells contrasts with the under-glycosylation observed for some recombinant glycoproteins transiently produced in *Nicotiana benthamiana* leaves [36,37]. It has recently been demonstrated that it is possible to further improve the N-glycan occupancy in plants by engineering the ER oligosaccharyltransferase complex [36], but this does not seem to be necessary in BY-2 cells.

By using two independent mass spectrometry methods, we were able to assess the overall glycan composition as well as the microheterogeneity at specific N-sites of the gB ectodomain produced in CHO cells. We showed that the glycosylation profile differed on each glycosite, some sites being occupied exclusively by oligomannose type N-glycans (N208, N281, N341) and others by complex bi- and tri-antennary N-glycans with a high level of galactosylation and sialylation. Theoretically, the distinction between oligomannose and complex type N-glycans gives information on the protein subcellular localization but, in our case, we analyzed a glycoprotein secreted in the culture medium which passed throughout the whole secretory pathway. The most probable explanation is that the accessibility of the three N-sites, N208, N281 and N341, to glycosylation-processing enzymes is sterically hampered. Furthermore, we showed that the glycosylation profile of gB expressed in BY-2 cells is similar to its counterpart produced in CHO cells, suggesting that the accessibility of the N-glycan to the processing machinery is preserved in CHO and BY-2 cells. However, the processed N-sites displayed higher homogeneity in BY-2 cells with a large proportion (30–69%) of the complex bi-antennary structure GnGnXF on N68, N73, N85, N286, N302, N405-N409, N452, N554, N585. On the contrary, in CHO-produced gB, there is an important proportion of highly processed complex N-glycans containing beta-1,4-galactose and sialic acid as compared to the relative low amount (<30%) of complex biantennary GnGnF. This could be due to a lower activity/amount of enzymes elongating the structure beyond GlcNAc residues in BY-2 cells. Eventually, it could be of interest to analyze the glycoprofile of the gB ectodomain produced in different culture conditions since a study on the N-linked glycosylation of human secreted alkaline phosphatase produced in tobacco NT1 suspension cells has shown that the relative abundance of GnGnXF, GnMXF and MMXF might be affected by the culture medium composition [10].

The gB protein analyzed in this study encompasses four out of the five characterized antigenic domains: AD-1 (540–640), AD2-I (68–77), AD5 (133–343) and AD4 (121–132, 344–438) [22]. The protein is heavily glycosylated and it is unknown whether production in CHO and BY-2 cells alters key antigenic regions or modifies the host innate immune response. Since these two phenomena can significantly affect the vaccine performance, they should be evaluated first by in vitro testing anti-gB antibodies that recognize different epitopes, then by in vivo immunological assays. The presence of the glycoepitopes beta(1,2)-xylose, alpha(1,3)-fucose epitopes and Lewis A extensions raises concerns of potential allergic reactions and rapid clearance following administration [38]. However, it is worth noting that the Medicago’s HA-based vaccine candidates transiently produced in wild type *N. benthamiana* leaves also displayed such structures [18] and were reported to be safe [39]. It would, however, be of interest to produce the gB construct in the BY-2 knocked-out lines recently obtained through CRIPSR/cas9 gene editing [14,40].

## 4. Materials and Methods

### 4.1. Expression of gB Ectodomain in BY-2 Cells

HCMV gB from strain AD169 (UniProtKB-P06473) was codon-optimized for expression in plant cells and synthesized (Genewiz) with flanking *Kpn*I and *Sac*I restriction sites. The gB construct comprises the endogenous signal peptide (residues 1–22) and the ectodomain and the membrane proximal regions (truncated between residues Ser64 and Ala724 to remove the transmembrane domain and the cytoplasmic tail) fused to a 6xHis-tag. The endogenous furin cleavage site Arg456ThrArgArg was replaced by Thr456ThrGlnThr. The *Kpn*I/*Sac*I fragment was placed under the control of the enhanced PMA4 promoter (En2pPMA4) and the NOS (nopaline synthase) terminator into the pAUX3131 plasmid, and the gB expression cassette was then introduced into the I-*Sce*I of the binary plasmid pPZP-nptII-mCherry, which contained a kanamycin resistance gene and an mCherry expression cassette [41].

Transformation of *N. tabacum* BY-2 cells was performed by biolistics. Transgenic calli were selected on kanamycin-containing medium and suspension cell lines were established by transferring calli into liquid Murashige and Skoog (MS) medium (MP Biomedicals, Solon, OH, USA). The extracellular medium of 20 transgenic cell lines grown in liquid culture (4 mL) was collected at early stationary phase (7 days old) by filtration on three layers of Miracloth (Calbiochem, Billerica, MA, USA).

For ELISA tests, high-binding microtiter plates (Greiner Bio-One) were coated with 100 µL/well goat anti-cytomegalovirus late matrix antigen (2.5 µg/mL) (BIORAD 2470-5004) in PBS (phosphate buffered saline) overnight at 4 °C. The plates were washed and blocked by adding 200 µL of PBST (PBS containing 0.05% Tween 20) containing 1% (*w*/*v*) BSA (bovine serum albumin) at room temperature for 1 h. After removing the blocking buffer, 100 µL/well serially dilutions in PBS of the extracellular medium samples were transferred to the ELISA plate and incubated at room temperature for 2 h. Serial dilutions of gB standard (produced in CHO cells) were added for calibration. The microtiter plates were then washed with PBST and incubated for 16 h at 4 °C with 100 µL/well (12.5 ng) rabbit anti-gB (Sino Biologicals #10202) diluted in PBS. The plates were washed and incubated for 1 h with 100 µL/well (33 ng) rat horseradish peroxidase HRP-conjugated anti-gamma heavy chain of rabbit IgGs (SynAbs LO-RG-1). After four washes in PBST, 100 µL of o-phenylenediamine (0.4 mg OPD/mL, SIGMA) peroxidase substrate in citrate buffer (0.02 M citric acid, 0.05 M Na_2_HPO_4_, pH 5.0) was added, then after 15 min at room temperature, 50 µL of stopping solution (5.5 M H_2_SO_4_) was added and the absorbance measured at 492 nm (SPECTROstar Nano, BMG Labtech, Ortenberg, Germany).

For protein analysis by Western blotting, extracellular medium samples (40 µL) or fractions eluted from size exclusion chromatography (40 µL) were solubilized at 100 °C for 5 min in SDS loading buffer with DTT (1,4-dithiothreitol), separated by SDS-PAGE (4–20% acrylamide), and stained with Colloidal Blue or transferred onto a polyvinylidene fluoride membrane, which was incubated with rabbit monoclonal antibody to human CMV gB (1:2,000, Sino Biological #10202-R038, Beijing, China) followed by anti-rabbit HRP antibodies (1:10,000, SynAbs LO-RG-1, Gosselies, Belgium) or anti-rabbit AP (IgG alkaline phosphatase) antibodies (1:10,000, Sigma-Aldrich A3687, St Louis, MO, USA).

### 4.2. Semi-Purification of gB from BY-2 Culture Medium

The transgenic gB line was grown in 50 mL of D11B medium [32] without cyclodextrin in two 250 mL Erlenmeyer flasks. The extracellular medium (70–80 mL) of 11 day old cultures was collected by filtration on three layers of Miracloth (Calbiochem) and centrifuged at 8000× *g* for 30 min. Solid (NH_4_)_2_SO_4_ was slowly added to the culture medium with stirring at room temperature to 60% saturation (28 g ammonium sulfate/80 mL). The solution was kept for 30 min at room temperature and then for 2 h at 4 °C. After centrifugation at 8000× *g* for 30 min at 4 °C, the supernatant (containing no gB protein detected by Western Blotting) was removed and the pellet was resuspended in 500 µL of buffer (20 mM Bicine, 50 mM NaCl, 10% glycerol, 1 mM PMSF (phenylmethanesulfonyl fluoride), protease inhibitor cocktail (1 µg/mL each of leupeptin, aprotinin, antipain, chymostatin and pepstatin), adjusted to pH 7.5 with NaOH). The sample was desalted on a PD-10 filtration column (GE Healthcare, Uppsala, Sweden) and clarified by centrifugation at 100,000× *g* for 15 min at 4 °C and then applied to size exclusion chromatography on Superdex 200 Increase 10/300 GL (GE Healthcare) equilibrated with buffer (20 mM Bicine, 50 mM NaCl, 10% glycerol, pH 7.5 (NaOH)) using the Akta Explorer 10 system (GE Healthcare) at a flow rate of 0.4 mL/min. Fractions of 1 mL were collected and analyzed by SDS-PAGE and Western blotting. CHO-purified gB (7 µg) was diluted in buffer (20 mM Bicine, 50 mM NaCl, 10% glycerol, pH 7.5 (NaOH)) and analyzed on Superdex 200 Increase 10/300 GL (GE Healthcare) following the same procedure. Before mass spectrometry analysis, the two fractions (F9-F10) containing the gB protein were pooled and concentrated 9.7 times using a centrifugal filter with a molecular weight cut-off of 3000 (Amicon Ultra-4 3K, Merck-Millipore, Cork, Ireland) and quantified using RC DC (reducing agent compatible and detergent compatible) Protein Assay (Bio-Rad, Hercules, CA, USA).

### 4.3. Expression and Purification of gB Ectodomain in CHO Cells

A stable clone expressing the gB protein (the same construct as described above in Section 4.1 for expression in BY-2 cells) generated with the CHOK1 SV cell line (Lonza Biologics, Slough, UK), was grown in suspension in CD-CHO medium (Invitrogen, Carlsbad, CA, USA) containing 50 µM L-Methionine sulfoximine (MSX; Sigma-Aldrich, St Louis, MO, USA) in the presence of 8% CO_2_, 140 rpm sub-passaged twice a week by dilution at 3 × 10^5^ cells/mL. For gB protein production, the clone was centrifuged (250× *g*—10 min) and seeded at 2 × 10^6^ cells/mL in CD-CHO medium for 6 days, at 29 °C (140 rpm—8% CO_2_).

After the incubation period, the cells were removed by centrifugation. The supernatant was implemented with 300 mM NaCl and protease inhibitor (Complete protease inhibitor cocktail tablets without EDTA, Roche) and loaded onto Ni Sepharose 6 Fast Flow (GE Healthcare) previously equilibrated in Buffer A (20 mM Bicine, 500 mM NaCl pH 8.3). The column was washed with three column volume (CV) of Buffer B (20 mM Bicine, 500 mM NaCl, 12.5 mM imidazole pH 8.3). The protein was eluted with Buffer C (20 mM Bicine, 500 mM NaCl, 500 mM imidazole pH 8.3) and the protein buffer was exchanged by gel filtration G25 (GE Healthcare) with Buffer A. The protein was loaded onto Ni HisTrap (GE Healthcare) previously equilibrated in Buffer A. The column was washed with three CV of Buffer A and the protein was eluted with three CV of Buffer C. The protein was further purified by size exclusion chromatography using a Superdex 200 26/60 (GE Healthcare) equilibrated in Buffer A. The fractions containing the protein of interest were pooled together and concentrated with a Vivaspin 20 (Sartorius, Goettingen, Germany) with a 30 kDa cut-off to reach a concentration of 0.5 mg/mL. The protein was then passed through a 0.22 µM filter and stored at −80 °C. The protein concentration was determined by RC DC Protein Assay (Biorad).

### 4.4. Glycoproteomic Workflow

Both gB samples (SEC-purified from CHO and BY-2 cells) were first reduced (10 mM dithiothreitol) for 40 min at 56 °C, alkylated (20 mM iodoacetamide) for 30 min at room temperature, and purified using a 2D Clean-Up kit (GE Healthcare). The proteins were then resuspended in 50 mM NH_4_HCO_3_ and proteolysis was next performed by a two-step trypsin treatment: 16 h at 37 °C at 1/50 ratio followed by 3 h at 37 °C at 1/100 ratio in 80% acetonitrile. The peptides were then dried.

A part of each sample was deglycosylated at 37 °C for 24 h using either PNGase F (8 U/100 µg, Roche) in 50 mM NH_4_HCO_3_ for gB produced in CHO cells or PNGase A (0.2 mU/100 µg, Roche, Basel, Switzerland) in a citrate 0.1 M/phosphate buffer adjusted to pH 5 for gB produced in BY-2 cells. PNGase A is able to cleave N-linked glycans with or without a core alpha(1,3)-fucose. PNGase A was not used for the deglycosylation of gB produced in CHO cells because it has been reported to be ineffective on sialylated N-Glycans [42], although PNGase A has been used to deglycosylate IgA and IgE produced in HEK293 cells [37,43]. These PNGase treatments deamidate asparagine into aspartic acid when asparagine is glycosylated. The ratio of deamidated to unmodified peptides upon-N-glycan release was used to evaluate the N-glycosylation site occupancy. The ionization efficiency bias is expected to be the same for gB derived from BY-2 and CHO-cells because identical peptides pairs were used.

A second digestion protocol was used in order to improve sequence coverage and increase the number of glycosites detected. The methodology applied relies on a Multiple Enzymatic Limited Digestion protocol (MELD) [35], consisting of a single two-hour proteolytic digestion at 37 °C involving the synergic action of a diluted mix of trypsin, chymotrypsin and Glu-C enzymes. This digestion engenders numerous missed cleavage events and results in abundant peptides of various length with overlapping stretches of residues allowing comprehensive protein characterization. Generated peptides were then analyzed by LC-MS/MS.

Peptides were separated by reverse-phase chromatography using Ultra Performance Liquid Chromatography (UPLC-MClass, HSS T3 column, Waters, Milford, MA, USA) in one dimension with an increasing ratio of acetonitrile/water (5–40% for 70 min) at a 600 nL/min flow rate. The chromatography system was coupled with a Thermo Scientific Q Exactive Hybrid Quadrupole-Orbitrap Mass Spectrometer (Thermo Fisher Scientific, Waltham, MA, USA), programed for data-dependent acquisition mode. Survey scans were acquired at 70,000 mass resolving power (full width at half maximum). An ion mass range from 400 to 1750 *m*/*z* was acquired in MS mode, and 3 × 10^6^ ions were accumulated in the survey scans. Ion trap Higher energy Collision Dissociation fragmentations at NCE (Normalized Collision Energy) 25 were performed within 2 amu isolation windows and a dynamic exclusion was enabled for 10 s.

### 4.5. Glycoproteomic Data Treatment

N-glycopeptides generated several reporter ions due to the preferential gas phase fragmentation of the polysaccharide part. The glycan oxonium ions [HexNAc]^+^ 204.09 and [HexNAc + Hex]^+^ 366.14 were used to identify the MS/MS spectra of all glycopeptides from the peak list (Table S3) Next, the presence of the Y1 ions (composed of the peptide linked to the first GlcNAc residue of the *N*-glycan) was also checked to sort out all MS/MS spectra for each glycosite. All potential Y1 ions were determined based on a prior theoretical digestion of the protein (allowing two missed cleavages for trypsin and seven missed cleavages in case of multienzymatic digestion). These two steps were performed using an in-house developed algorithm and MS/MS spectra were then manually curated. For the assigned spectra, the composition of the glycopeptides was determined based on their precursor mass using the GlycoMod tool (available at http://web.expasy.org/glycomod/) and their MS/MS spectra. Following that step, the relative abundance of each glycoform was determined by integration of MS1 chromatograms using Skyline 3.1. software (MacCoss Lab Software, University of Washington, Seattle, WA, USA).

Deglycosylated peptides were analyzed to determine occupancy of the N-glycosylation sites. For every detectable site (based on a database search performed using Mascot search engine), MS1 chromatograms of unoccupied (containing an Asn residue) and occupied (containing a neoformed Asp residue) peptides were integrated using Skyline 3.1 and compared.

### 4.6. N-Glycosylation Profile of gB Produced in CHO Cells

In order to assess the N-glycan diversity of CHO-produced gB, N-glycans were purified, after PNGase F treatment filled with NaOH beads according to a procedure adapted from [44]. Briefly, N-glycans were resuspended in 30 µL DMSO and 20 µL methyl iodide was added. The mixture was incubated for 30 min. Samples were then centrifugated and 20 µL of methyl iodide was added. The sample was then reloaded and incubated at room temperature for 20 min. The sample was recovered by centrifugation and the column was washed twice with 100 µL acetonitrile. The sample was eventually purified using liquid–liquid extraction in chloroform. After solvent evaporation, samples were resuspended in 50/50 H_2_O/ACN and spotted on a MALDI plate with an equivolume of 2.5 DHB (2,5-dihydroxybenzoic acid) matrix (20 mg/mL in 50/50 H_2_O/ACN). Samples were analyzed on a MALDI-TOF/TOF mass spectrometer (Ultraflextreme, Bruker, Bremen, Germany) in positive reflectron mode and data were interpreted using FlexAnalysis 3.4 software (Bruker).

## Figures and Tables

**Table 1:** N-Glycosylation site occupancy of gB produced in CHO and BY-2. N-glycosylation site occupancy (%) was deduced from the ratio of deamidated to unmodified peptide upon N-glycan release with PNGase F (CHO) or PNGase A (BY-2). ND: not detected.

N-Glycosite	Peptides Identified after PNGase	N-Glycan Occupancy	CHO	BY-2
N68-N73	ANETIYNTTLK	N68-N73 non-glycosylated	0.7	2.9
	ADETIYNTTLK	N68 glycosylated/N73 non-glycosylated	56.5	43.4
	ANETIYDTTLK	N68 non-glycosylated/N73 glycosylated	3.2	3.5
	ADETIYDTTLK	N68-N73 glycosylated	39.7	50.2
N85	YGDVVGVNTTK	N85 non-glycosylated	2.3	ND
	YGDVVGVDTTK	N85 glycosylated	97.7	100
N286	NASYFGENADK	N286 non-glycosylated	9.7	ND
	DASYFGENADK	N286 glycosylated	90.3	100
N383	QEVNMSDSALDCVR	N383 non-glycosylated	4.3	3.2
	QEVDMSDSALDCVR	N383 glycosylated	95.7	96.8
N405-N409	LQQIFNTSYNQTYEK	N405-N409 non-glycosylated	0.3	ND
	LQQIFDTSYNQTYEK	N405 glycosylated/N409 non-glycosylated	ND	ND
	LQQIFNTSYDQTYEK	N405 non-glycosylated/N409 glycosylated	28.5	36.4
	LQQIFDTSYDQTYEK	N405-N409 glycosylated	71.1	63.6
N208-N302-N341-N417-N452-N554-N585	NXS/T peptides	non-glycosylated	ND	ND
	DXS/T peptides	glycosylated	100	100

## Supplementary Materials

Supplementary materials can be found at https://www.mdpi.com/1422-0067/20/15/3741/s1. Figure S1: Nomenclature of N-glycans structures referred to, Figure S2: Annotated MS/MS spectra of the (degly)cosylated peptides containing N452 and N464-N465, Table S1: Distribution of the N-glycans detected on CHO-produced gB, Table S2: Relative abundance of glycans on each N-glycosite, Table S3: Oxonium ions.
