# Supplementary material for: Exploring the N-Glycosylation Profile of Glycoprotein B from Human Cytomegalovirus Expressed in CHO and Nicotiana tabacum BY-2 Cells
Source: Int J Mol Sci. 2019 Jul 31;20(15):3741. doi: 10.3390/ijms20153741 (PMC6696289; doi:10.3390/ijms20153741)
Supplement: Supplementary file 1 [file ijms-20-03741-s001.pdf]

Figure S1. Nomenclature of N-glycans structures referred to .
N-Glycans are abbreviated according to the ProGlycAn system http://www.proglycan.com/protein-glycosylation-analysis/nomenclature. Lea: Lewis A structure.
The symbols for the monosaccharides are drawn according to the nomenclature from the Consortium for Functional Glycomics: N-acétylglucosamine; fucose; galactose; mannose; xylose; N-acetylneuraminidic acid

A. Structures observed in CHO and plant cells

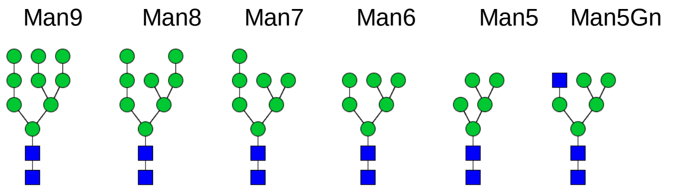

B. Structures observed in plant cells

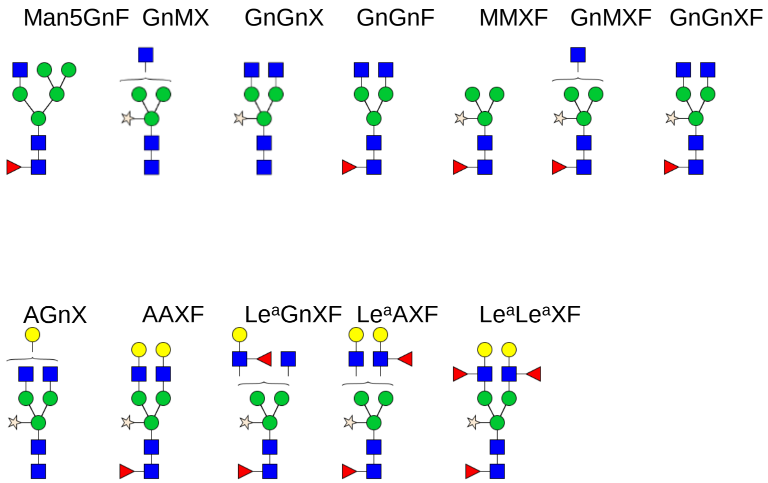

C. Structures observed in CHO cells

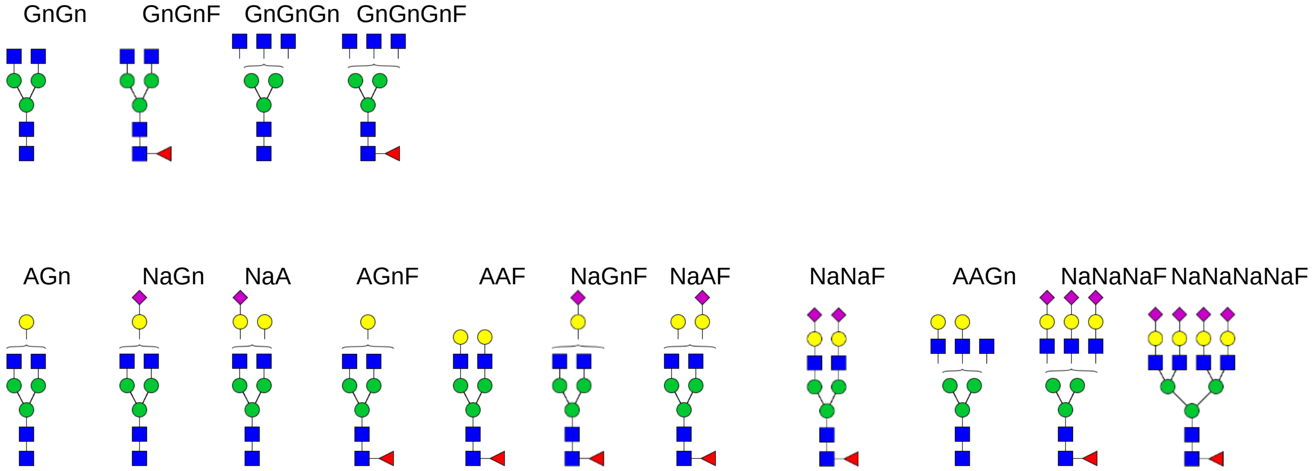

Figure S2.

A. Annotated MS/MS spectrum of the deglycosylated peptide containing N452.
Y7 ion shows unambiguously the presence of a deamidated asparagine residue, resulting from deglycosylation process.

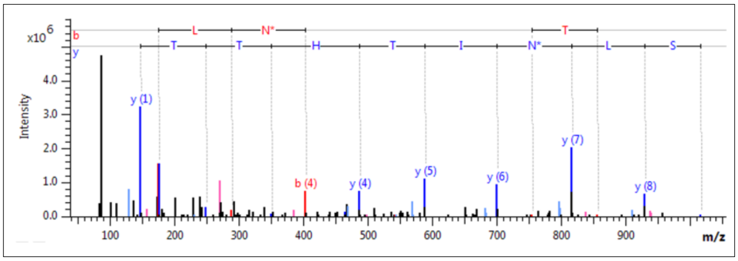

B. Annotated MS/MS spectrum of the glycopeptide containing N452 decorated with the glycan NaNaF.
As expected, Y1 ion (P+Gn) predominates in the glycopeptide fragments. Its m/z value demonstrates the presence of N452 peptidic sequence as no other gB glycosite would generate such m/z.

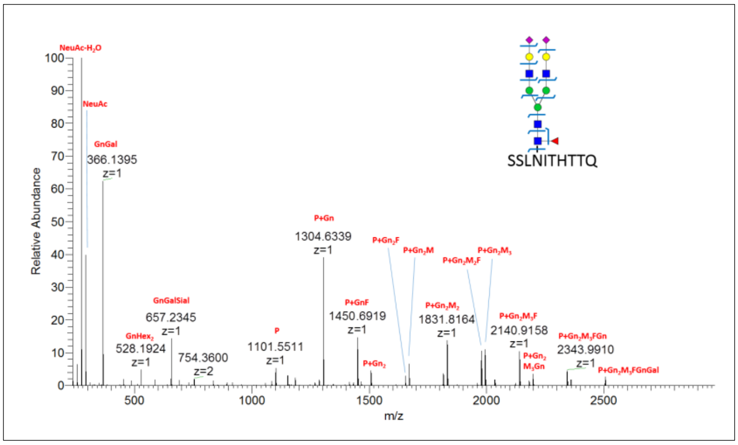

C. Annotated MS/MS spectrum of the deglycosylated peptide containing N464-N465.
Y6 ion shows unambiguously the presence of a deamidated asparagine residue, resulting from deglycosylation process of N464.

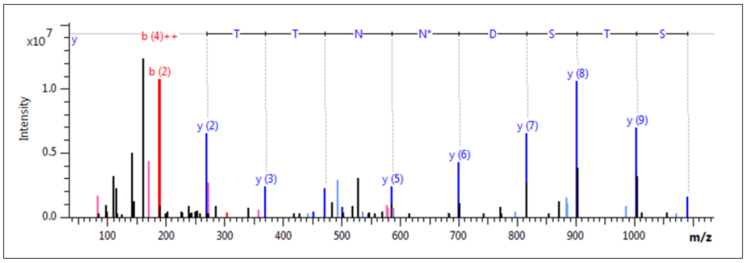

D. Annotated MS/MS spectrum of the glycopeptide containing N464-N465 decorated with the glycan NaAF.
As expected, Y1 ion (P+Gn) predominates in the glycopeptide fragments. Its m/z demonstrates the presence of N452 peptidic sequence as no other gB glycosite would generate such m/z.

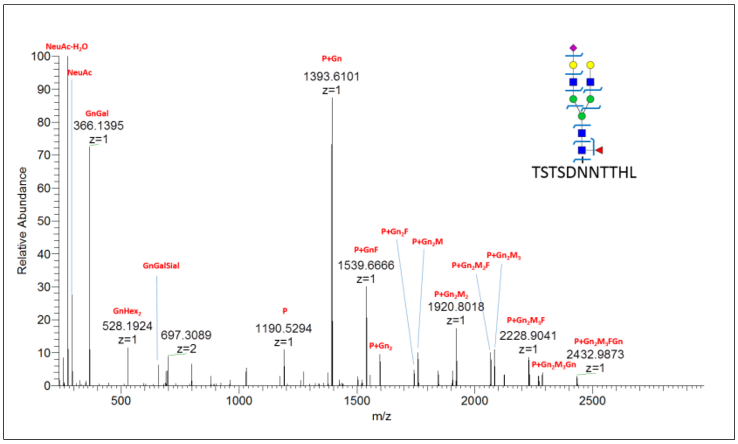

Supplementary Table 1. Distribution of the N-glycans detected on CHO-produced gB

m/z	glycan	Area (%)
1375.72	$HexNAc_2Hex_4$	0.8
1579.82	HexNAc2Hex5	4.7
1590.83	$HexNAc_3Hex_3Fuc$	1.0
1620.85	$HexNAc_3Hex_4$	0.5
1661.87	HexNAc4Hex3	2.3
1783.91	HexNAc2Hex6	3.4
1794.92	$HexNAc_3Hex_4Fuc$	0.4
1824.93	$HexNAc_3Hex_5$	0.7
1835.95	HexNAc4Hex3Fuc	11.8
1865.96	$HexNAc_4Hex_4$	1.8
1988.00	HexNAc2Hex7	3.0
2040.05	HexNAc4Hex4Fuc	4.8
2070.04	$HexNAc_4Hex_5$	1.2
2081.08	$HexNAc_5Hex_3Fuc$	2.7
2192.10	HexNAc2Hex8	3.0
2227.13	$HexNAc_4Hex_4NeuAc$	2.1
2244.15	HexNAc4Hex5Fuc	3.4
2285.17	$HexNAc_5Hex_4Fuc$	0.9
2396.20	$HexNAc_2Hex_9$	1.0
2401.21	HexNAc4Hex4FucNeuAc	9.6
2431.23	HexNAc4Hex5NeuAc	2.8
2489.28	$HexNAc_5Hex_5Fuc$	0.9
2605.32	HexNAc4Hex5FucNeuAc	10.1
2635.30	$HexNAc_4Hex_6NeuAc$	0.8
2646.34	$HexNAc_5Hex_4FucNeuAc$	1.1
2676.33	$HexNAc_3Hex_5NeuAc$	0.2
2693.35	$HexNAc_5Hex_6Fuc$	0.8
2792.40	$HexNAc_4Hex_5NeuAc_2$	0.6
2850.42	$HexNAc_5Hex_5FucNeuAc$	0.7
2880.44	$HexNAc_5Hex_6NeuAc$	0.2
2966.49	HexNAc4Hex5FucNeuAc2	8.0
2996.51	$HexNAc_4Hex_6NeuAc_2$	0.8
3054.55	HexNAc5Hex6FucNeuAc	1.5
3071.541	HexNAc5Hex7Fuc2	0.4
3084.54	$HexNAc_5Hex_6FucNeuGc$	0.2
3130.53	$HexNAc_5Hex_7NeuGc$	0.2
3197.60	$HexNAc_5Hex_4Fuc_2NeuAc_2$	0.1
3211.63	HexNAc5Hex5FucNeuAc2	0.9
3241.64	$HexNAc_5Hex_6NeuAc_2$	0.1
3387.71	$HexNAc_7Hex_7Fuc$	0.1
3401.71	$HexNAc_5Hex_5Fuc_2NeuAc_2$	0.2
3415.74	HexNAc5Hex6FucNeuAc2	1.8

3445.73	$HexNAc_4Hex_7NeuAc_2$	0.1
3456.76	$HexNAc_6Hex_5FucNeuAc_2$	0.1
3503.74	$HexNAc_6Hex_7FucNeuAc$	0.1
3602.82	HexNAc5Hex6NeuAc3	0.1
3660.84	$HexNAc_6Hex_6FucNeuAc_2$	0.1
3776.90	HexNAc5Hex6FucNeuAc3	3.8
3806.92	$HexNAc_5Hex_6FucNeuAc_2NeuGc$	0.6
3865.01	$HexNAc_6Hex_7FucNeuAc_2$	0.7
3882.05	$HexNAc_6Hex_8Fuc_2NeuAc_1$	0.2
3894.76	$HexNAc_6Hex_8NeuAc_2$	0.1
4022.06	$HexNAc_6Hex_6FucNeuAc_3$	0.1
4226.12	HexNAc6Hex7FucNeuAc3	0.2
4314.20	$HexNAc_7Hex_8FucNeuAc_2$	0.0
4587.29	HexNAc6Hex7FucNeuAc4	1.5
4617.35	$HexNAc_6Hex_7FucNeuAc_3NeuGc_1$	0.1
4675.44	$HexNAc_7Hex_8FucNeuAc_3$	0.1
4705.33	$HexNAc_7Hex_9NeuAc_3$	0.1
		99.6

The glycans highlighted in green are represented in Figure 4

CHO

Glycosite N68 (peptide SHRANETIY-M)	
Glycan	Relative Abundance (%)
Gn2M3Gn2F1Hex2NeuAc2	28.7
Gn2M3Gn2F1Hex2NeuAc1	16.2
Gn2M3Gn2F1Hex1NeuAc1	15.6
Gn2M3Gn2F1	11.2
Gn2M3Gn2F1Hex1	5.2
Gn2M3Gn3F1Hex3NeuAc2	3.9
Gn2M3Gn1F1	2.9
Gn2M3Gn3F1	2.1
Gn2M3Gn3F1Hex1NeuAc1	1.9
Gn2M3Gn3F1Hex2NeuAc2	1.7
Gn2M3Gn2F1Hex2NeuAc1NeuGc1	1.6
Gn2M3Gn3F1Hex3NeuAc1	1.2
Gn2M3Gn3F1Hex2NeuAc1	1.0
Gn2M3Gn1F1Hex1NeuAc1	0.8
Gn2M3Gn3F1Hex1	0.7
Gn2M3Gn2Hex2NeuAc1	0.6
Gn2M3Gn1F1Hex1	0.5
Gn2M3Gn4F1Hex4NeuAc2	0.5
Gn2M3Gn4F1Hex2NeuAc2	0.5
Gn2M3Gn2Hex3NeuAc1	0.5
Gn2M3Gn4F1Hex1NeuAc1	0.4
Gn2M3Gn3F1Hex2	0.3
Man5	0.3
Gn2M3F1	0.3
Gn2M3Gn4F1	0.3
Gn2M3Gn3F1Hex3	0.3
Gn2M3Gn2F1Hex3NeuAc1	0.2
Gn2M3Gn1F2Hex1NeuAc2	0.1
Gn2M3Gn2Hex1NeuAc1	0.1
Gn2M3Gn3F1Hex2NeuAc1NeuGc1	0.1
Gn2M3Gn2Hex1	0.1
High Mannose	0.4
Complex (Gn)(Gn)(F)	14.3
Complex longer than GnGnXF including NeuAc	85.3

BY2

Glycosite N68 (peptide SHRANETIY-M)	
Glycans	Relative Abundance (%)
Gn2M3Gn2FX	35.3
Gn2M3Gn2F3XHex2	25.5
Gn2M3Gn2F2XHex	17.6
Gn2M3GnFX	9.2
Gn2M3Gn2F2XHex2	4.2
Gn2M3Gn2FXHex	1.9
Gn2M3Gn2F2XHex3	1.7
Gn2M3Gn2FXHex2	1.6
Gn2M3Gn2XHex	1.5
Gn2M3FX	0.5
Gn2M3GnF2XHex	0.5
Gn2M3GnXHex	0.3
High mannose	0
Complex (Gn)(Gn)(X)(F)	62.6
Complex longer than GnGnXF including Lewis	37.4

CHO

Glycosite N73 (peptide TIYNTTLK-M)	
Glycan	Relative Abundance (%)
Gn2M3Gn2F1	18.9
Gn2M3Gn2F1Hex2NeuAc1	17.8
Gn2M3Gn2F1Hex1NeuAc1	16.5
Gn2M3Gn2F1Hex1	8.1
Gn2M3Gn3F1	7.7
Man5	5.7
Gn2M3Gn3F1Hex3NeuAc2	4.4
Gn2M3Gn3F1Hex3NeuAc1	3.4
Gn2M3Gn2F1Hex2	3.3
Gn2M3Gn1F1	3.2
Gn2M3Gn3F1Hex1NeuAc1	2.5
Gn2M3Gn3F1Hex2NeuAc1	2.0
Gn2M3Gn3F1Hex1	1.5
Man6	1.5
Gn2M3Gn1	1.3
Gn2M3Gn2F1Hex1NeuGc1	1.1
Gn2M3Gn2Hex1NeuAc1	0.8
High Mannose	7.2
Complex (Gn)(Gn)(F)	23.5
Complex longer than GnGnXF including NeuAc	69.3

BY2

Glycosite N73 (peptide TIYNTTLK-M)	
Glycans	Relative Abundance (%)
Gn2M3Gn2FX	61.6
Gn2M3GnFX	11.5
Gn2M3Gn2F3XHex2	9.5
Gn2M3Gn2F2XHex	6.4
Gn2M3Gn2F2XHex2	4.0
Gn2M3Gn2FXHex	1.8
Gn2M3GnFXHex	1.7
Gn2M3Gn2XHex	1.2
Gn2M3Gn2FXHex2	1.0
Gn2M3GnF2XHex	0.4
Gn2M3FX	0.3
Man5	0.3
Man7	0.2
Gn2M3GnF2XHex2	0.2
High mannose	0.6
Complex (Gn)(Gn)(X)(F)	73.3
Complex longer than GnGnXF including Lewis	26.1

CHO

Glycosite N85 (peptide YGDVVGVNTTK-T)	
Glycan	Relative Abundance (%)
Gn2M3Gn2F1	12.3
Gn2M3Gn2F1Hex2	10.7
Gn2M3Gn2F1Hex2NeuAc2	10.3
Gn2M3Gn2F1Hex2NeuAc1	10.0
Gn2M3Gn2F1Hex1	9.4
Man5	7.8
Gn2M3Gn2F1Hex1NeuAc1	6.9
Man6	5.2
Gn2M3Gn2Hex2NeuAc1	3.0
Gn2M3Gn2Hex3NeuAc2	2.5
Gn2M3Gn2	2.4
Man7	2.3
Gn2M3Gn1Hex2	2.2
Man8	1.9
Gn2M3Gn2Hex1	1.8
Gn2M3Gn2Hex1NeuAc1	1.4
Gn2M3Gn2Hex2	1.4
Gn2M3Gn2Hex2NeuAc2	1.0
Gn2M3	1.0
Gn2M3Gn1	0.9
Gn2M3Gn1F1	0.8
Gn2M3Gn3F1Hex3	0.7
Gn2M3Gn1F1Hex1	0.7
Gn2M3Gn1Hex3NeuAc1	0.6
Gn2M3Gn3F1Hex1NeuAc1	0.5
Gn2M3Gn1Hex2NeuAc1	0.5
Gn2M3Gn1F1Hex1NeuAc1	0.5

BY2

Glycosite N85 (peptide YGDVVGVNTTK-T)	
Glycans	Relative Abundance (%)
Gn2M3Gn2FX	42.3
Gn2M3GnFXHex	17.8
Gn2M3Gn2FXHex2	7.5
Gn2M3Gn2F2XHex	7.4
Gn2M3Gn2F3XHex2	6.7
Gn2M3GnFX	4.3
Man6	3.8
Man7	3.0
Man5	2.4
Gn2M3GnFHex2	2.0
Man8	1.2
Gn2M3FXHex	0.7
Gn2M3GnFHex	0.4
Gn2M3FX	0.4
High mannose	10.3
Complex (Gn)(Gn)(X)(F)	47.8
Complex longer than GnGnXF including Lewis	41.9

Gn2M3Gn1Hex1NeuAc1	0.5
Gn2M3Gn1Hex1	0.3
Gn2M3Gn1F2NeuAc1	0.1
Gn2M3Gn1F1Hex3	0.1
Gn2M3F1	0.1
Gn2M3F1Hex5	0.1
High Mannose	46.3
Complex (Gn)(Gn)(F)	36.7
Complex longer than GnGnXF including NeuAc	16.1

CHO

Glycosite N208 (peptide VAYHRDSYENK-M)

Glycan	Relative Abundance (%)
Man5	17.3
Man9	14.0
Man8	12.4
Man6	11.4
Man7	10.5
Gn2M3Hex1	5.4
Gn2M3	3.7
Gn2M3Gn2F1Hex1	2.6
Gn2M3Gn2F1	2.6
Gn2M3Gn2F1Hex2NeuAc1	2.5
Gn2M3Gn2F1Hex2NeuAc2	2.1
Gn2M3Gn1Hex1	2.0
Gn2M3Hex7	1.8
Gn2M3Gn2F1Hex2	1.7
Gn2M3Gn2	1.7
Gn2M3Gn2Hex1	1.6
Gn2M3Gn1Hex2	1.6
Gn2M3Gn2Hex2	1.3
Gn2M3Gn2F1Hex1NeuAc1	1.3
Gn2M3Gn1	1.3
Gn2M3Gn1F1	0.4
Gn2M3Gn2Hex1NeuAc1	0.4
Gn2M3Gn1F1Hex1	0.4
Gn2M3Gn1Hex2NeuAc1	0.3
High Mannose	76.4
Complex (Gn)(Gn)(F)	5.9
Complex longer than GnGnXF including NeuAc	17.6

BY2

Glycosite N208 (peptide VAYHRDSYENK-M)

Glycans	Relative Abundance (%)
Man9	47.8
Man7	26.0
Man5	9.3
Gn2M3GnX	8.9
Gn2M3Gn2FX	8.0
High mannose	83.1
Complex (Gn)(Gn)(X)(F)	16.9
Complex longer than GnGnXF including Lewis	0.0

CHO

Glycosite N281 (peptide SKYPYHFFATSTGDVVYISPFYNGTNR-T)

Glycan	Relative Abundance (%)
Man9	38.2
Man8	24.3
Man7	13.0
Man4	10.1
Man6	7.3
Man5	7.1
High Mannose	100.0
Complex (Gn)(Gn)(F)	0.0
Complex longer than GnGnXF including NeuAc	0.0

BY2

Glycosite N281 (peptide YNGTNR-M)

Glycans	Relative Abundance (%)
Man8	41.0
Man7	25.5
Man9	17.7
Man6	12.2
Man5	3.7
High mannose	100
Complex (Gn)(Gn)(X)(F)	0
Complex longer than GnGnXF including Lewis	0

CHO

Glycosite N286 (peptide NASYFGENADK-T)

Glycan	Relative Abundance (%)
Gn2M3Gn2F1Hex2NeuAc1	22.2
Gn2M3Gn2F1	17.8
Gn2M3Gn2F1Hex1NeuAc1	14.0
Gn2M3Gn2F1Hex2NeuAc2	10.5
Gn2M3Gn2F1Hex1	8.8
Gn2M3Gn2F1Hex2	5.1
Gn2M3Gn3F1	3.8
Gn2M3Gn2Hex2NeuAc1	3.0
Gn2M3Gn2F2Hex3	3.0
Gn2M3Gn2	2.4
Gn2M3Gn1F1	1.6
Gn2M3Gn3F1Hex3NeuAc2	1.4
Gn2M3Gn2Hex1	1.3
Gn2M3Gn2Hex1NeuAc1	1.1
Man6	1.0
Man5	0.8
Gn2M3Gn2Hex2	0.7
Gn2M3Gn2F1Hex1	0.7
Gn2M3	0.4
Gn2M3Gn1Hex1	0.4
High Mannose	2.2
Complex (Gn)(Gn)(F)	22.2
Complex longer than GnGnXF including NeuAc	75.5

BY2

Glycosite N286 (peptide NASYFGENADK-T)

Glycans	Relative Abundance (%)
Gn2M3Gn2FX	66.3
Gn2M3Gn2F2XHex	9.7
Gn2M3Gn2F3XHex2	7.8
Gn2M3Gn2XHex	6.6
Gn2M3GnFX	5.3
Gn2M3Gn2F2XHex2	1.3
Gn2M3Gn2FXHex	1.1
Gn2M3GnFXHex	0.6
Gn2M3GnXHex	0.5
Gn2M3GnF2XHex	0.4
Gn2M3Gn2X	0.2
Gn2M3FX	0.2
High mannose	0.0
Complex (Gn)(Gn)(X)(F)	71.9
Complex longer than GnGnXF including Lewis	28.1

CHO

Glycosite N302 (peptide FFIFPNYTIVSDFGRPNQQPETHR-T)

Glycan	Relative Abundance (%)
Gn2M3Gn2F1	26.4
Gn2M3Gn2F1Hex1NeuAc1	18.1
Gn2M3Gn2F1Hex2NeuAc1	12.4

BY2

Glycosite N302 (peptide FFIFPNYTIVSDFGRPNQQPETHR-T)

Glycans	Relative Abundance (%)
Gn2M3Gn2FX	69.3
Gn2M3Gn2F2XHex	10.8
Gn2M3GnFX	9.4

Gn2M3Gn2F1Hex1	11.5
Gn2M3Gn2F1Hex2NeuAc2	7.9
Man5	5.1
Gn2M3Gn2F1Hex2	4.7
Gn2M3Gn2	4.5
Gn2M3Gn1F1	3.1
Man6	1.8
Gn2M3Gn2Hex1NeuAc1	1.6
Gn2M3Gn1	1.6
Gn2M3Gn2Hex1	1.4
High Mannose	6.9
Complex (Gn)(Gn)(F)	35.6
Complex longer than GnGnXF including NeuAc	57.6

Gn2M3Gn2F3XHex2	2.6
Gn2M3Gn2FXHex	2.1
Gn2M3Gn2XHex	1.6
Gn2M3Gn2F2XHex2	1.0
Gn2M3GnFXHex	1.0
Gn2M3Gn2FXHex2	0.6
Gn2M3FX	0.4
Gn2M3GnF2XHex	0.3
Gn2M3GnFXHex2	0.3
Gn2M3GnFXHex3	0.3
Gn2M3Gn2X	0.3
Gn2M3Gn2F	0.2
High mannose	0.0
Complex (Gn)(Gn)(X)(F)	79.5
Complex longer than GnGnXF including Lewis	20.5

CHO

Glycosite N341 (peptide NVTCQLTFWEASER-T)

Glycan	Relative Abundance (%)
Man9	27.1
Man8	24.5
Man7	12.6
Man5	12.0
Man6	11.4
Gn2M3Hex1	9.3
Gn2M3Gn1	3.1
High Mannose	96.9
Complex (Gn)(Gn)(F)	3.1
Complex longer than GnGnXF including NeuAc	0.0

BY2

Glycosite N341 (peptide NVTCQLTFWEASER-T)

Glycans	Relative Abundance (%)
Man6	29.2
Man7	12.1
Man5	10.6
Gn2M3Gn2FX	9.9
Gn2M3GnFX	8.4
Gn2M3GnXHex	6.7
Gn2M3GnHex2	6.5
Gn2M3GnX	6.4
Gn2M3GnFXHex	5.2
Gn2M3GnXHex2	2.8
Gn2M3GnFXHex2	1.2
Gn2M3GnHex	0.7
Man8	0.3
High mannose	52.2
Complex (Gn)(Gn)(X)(F)	31.2
Complex longer than GnGnXF including Lewis	16.5

CHO

Glycosite N383 (peptide QEVNMSDSALDCVR-T)

Glycan	Relative Abundance (%)
Gn2M3Gn2F1	14.9
Man7	12.8
Gn2M3Gn2F1Hex1NeuAc1	10.5
Gn2M3Gn3F1	9.4
Gn2M3Gn2F1Hex2NeuAc1	6.9
Man6	6.4
Gn2M3Gn2	6.4
Man9	6.3
Gn2M3Gn2F1Hex1	6.2
Man5	6.1
Gn2M3Gn1Hex2	4.8
Gn2M3Gn1	3.3
Gn2M3Gn1Hex1	2.9
Gn2M3Gn1Hex1NeuAc1	1.7
Gn2M3Gn3Hex3NeuAc1	1.1
High Mannose	31.7
Complex (Gn)(Gn)(F)	32.5
Complex longer than GnGnXF including NeuAc	35.8

BY2

Glycosite N383 (peptide KQEVNMSDSALDCVR-T)

Glycans	Relative Abundance (%)
Man7	26.3
Man8	18.9
Gn2M3GnFX	10.0
Gn2M3Gn2FX	9.3
Gn2M3GnFHex2	5.9
Gn2M3GnFXHex	5.1
Man6	3.9
Gn2M3GnFHex3	3.5
Gn2M3GnF2XHex	2.6
Gn2M3GnFHex	2.4
Gn2M3Gn2F	1.8
Man9	1.4
Gn2M3GnFXHex2	1.2
Gn2M3GnF2XHex2	1.1
Gn2M3GnF	0.9
Gn2M3GnF2Hex3	0.9
Gn2M3Gn2F2XHex	0.8
Gn2M3Gn2F3XHex2	0.7
Gn2M3Gn2F2XHex2	0.7
Gn2M3GnFXHex3	0.5
Gn2M3GnFHex4	0.4
Gn2M3Gn2FXHex2	0.4
Gn2M3GnXHex	0.3
Gn2M3GnF2Hex4	0.3
Gn2M3Gn2FXHex	0.3
Gn2M3Gn2F3Hex2	0.2
Gn2M3GnF2XHex3	0.1
Gn2M3GnX	0.1
Gn2M3Gn2F2Hex	0.1
High mannose	50.5
Complex (Gn)(Gn)(X)(F)	22.1
Complex longer than GnGnXF including Lewis	27.4

CHO

Glycosite N405-409 (peptide LQQIFNTSYNQTYEK-T)

Glycan	Relative Abundance (%)
Gn2M3Gn2F1Hex2NeuAc1	46.3
Gn2M3Gn2F1Hex1NeuAc1	33.9
Gn2M3Gn2Hex2NeuAc1	19.9
High Mannose	0.0
Complex (Gn)(Gn)(F)	0.0
Complex longer than GnGnXF including NeuAc	100.0

BY2

Glycosite N405-409 (peptide LQQIFNTSYNQTYEK-T)

Glycans	Relative Abundance (%)
Gn2M3Gn2FX	65.1
Gn2M3Gn2F3XHex2	8.2
Gn2M3GnFX	7.8
Gn2M3Gn2F2XHex	6.3
Gn2M3Gn2FXHex2	4.1
Gn2M3Gn2F2XHex2	2.8
Gn2M3GnFXHex	1.7
Gn2M3Gn2FXHex	1.4
Gn2M3FX	1.3
Gn2M3GnF2XHex	0.7
Gn2M3GnHex4	0.3
Gn2M3GnFHex3	0.2
Gn2M3GnF2XHex2	0.2

High mannose	0.0
Complex (Gn)(Gn)(X)(F)	74.7
Complex longer than GnGnXF including Lewis	25.3

CHO

Glycosite N417 (peptide YGNVSVFE-M)	
Glycan	Relative Abundance (%)
Man5	11.4
Man6	11.0
Gn2M3Gn2Hex2NeuAc1	9.0
Man7	8.4
Gn2M3Gn2F1Hex2NeuAc1	7.8
Man8	6.6
Gn2M3Gn2F1Hex2NeuAc2	5.7
Gn2M3Gn2	5.0
Gn2M3Gn2F1Hex1NeuAc1	4.9
Gn2M3Gn2Hex1NeuAc1	4.6
Gn2M3Gn3F1Hex3NeuAc3	4.2
Gn2M3Hex1	3.9
Gn2M3	3.0
Gn2M3Gn2F1	2.4
Gn2M3Gn3F1Hex3NeuAc1	2.2
Gn2M3Gn2Hex2	2.1
Gn2M3Gn2Hex1	2.0
Gn2M3Gn1Hex1NeuAc1	1.7
Gn2M3Gn2F1Hex2	1.0
Gn2M3Gn1Hex2	0.9
Gn2M3Gn3Hex3NeuAc1	0.9
Gn2M3Gn3F1Hex3	0.7
Gn2M3Gn3F1	0.7
High Mannose	44.3
Complex (Gn)(Gn)(F)	8.3
Complex longer than GnGnXF including NeuAc	47.4

BY2

Glycosite N417 (peptide kYGNVSVFE-M)	
Glycans	Relative Abundance (%)
Gn2M3Gn2FX	22.6
Man8	16.2
Man7	14.1
Gn2M3Gn2F	11.5
Gn2M3GnFX	7.1
Man6	7.1
Man5	6.4
Gn2M3Gn2F2XHex2	3.2
Gn2M3GnFXHex	2.7
Gn2M3GnFHex	2.4
Gn2M3GnFHex2	1.8
Gn2M3GnF	1.6
Gn2M3Gn2F2Hex2	0.9
Gn2M3Gn2F3Hex2	0.8
Gn2M3FX	0.8
Gn2M3GnF2XHex	0.7
High mannose	43.8
Complex (Gn)(Gn)(X)(F)	43.7
Complex longer than GnGnXF including Lewis	12.6

CHO

Glycosite N452 (peptide SSLNITHTTQ-M)	
Glycan	Relative Abundance (%)
Gn2M3Gn2F1	20.0
Gn2M3Gn2F1Hex2NeuAc2	18.4
Gn2M3Gn2F1Hex1NeuAc1	16.4
Gn2M3Gn2F1Hex2NeuAc1	12.1
Gn2M3Gn3F1	10.7
Gn2M3Gn2F1Hex1	5.0
Gn2M3Gn3F1Hex3NeuAc2	4.4
Gn2M3Gn3F1Hex1NeuAc1	4.4
Man5	3.1
Gn2M3Gn1F1	2.2
Gn2M3Gn3F1Hex3NeuAc1	2.1
Gn2M3Gn3F1Hex1	1.2
High Mannose	3.1
Complex (Gn)(Gn)(F)	22.2
Complex longer than GnGnXF including NeuAc	74.7

BY2

Glycosite N452 (peptide SSLNITHTTQ-M)	
Glycans	Relative Abundance (%)
Gn2M3Gn2FX	63.6
Gn2M3Gn2F3XHex2	28.8
Gn2M3GnFX	7.6
High mannose	0.0
Complex (Gn)(Gn)(X)(F)	71.2
Complex longer than GnGnXF including Lewis	28.8

CHO

Glycosite N464-465 (peptide TSTSDNNTHL-M)	
Glycan	Relative Abundance (%)
Gn2M3Gn3	16.9
Gn2M3Gn2Hex1	15.0
Gn2M3Gn3Hex2	11.3
Gn2M3Gn2F1	9.3
Gn2M3Gn2F1Hex1NeuAc1	8.1
Gn2M3Gn2F1Hex2NeuAc1	7.4
Gn2M3Gn2F1Hex1	5.4
Gn2M3Gn3F1	4.7
Gn2M3Gn2F1Hex2NeuAc2	4.6
Gn2M3Gn2F1Hex2	3.2
Gn2M3Gn3F1Hex1NeuAc1	2.7
Gn2M3Gn2	2.7
Gn2M3Gn3F1Hex1	1.1
Gn2M3Gn3F1Hex3NeuAc1	1.1
Gn2M3Gn3F1Hex3	1.0
Man6	1.0
Man7	1.0
Gn2M3Gn1Hex2	0.9
Man5	0.8
Gn2M3Gn1F1	0.8
Gn2M3Gn1Hex1	0.5
Gn2M3Gn1	0.3
High Mannose	2.9
Complex (Gn)(Gn)(F)	14.5
Complex longer than GnGnXF including NeuAc	82.6

CHO

Glycosite N554 (peptide TFMGDVLGLASCVTINQTSVK-T)	
Glycan	Relative Abundance (%)
Man5	11.7
Gn2M3Gn2F1Hex2NeuAc1	10.6
Gn2M3Gn2Hex2NeuAc1	9.3

BY2

Glycosite N554 (peptide TFMGDVLGLASCVTINQTSVK-T)	
Glycans	Relative Abundance (%)
Gn2M3Gn2FX	32.8
Gn2M3GnFX	22.1
Gn2M3GnFXHex	11.6

Gn2M3Gn2F1	8.9
Gn2M3Gn2F1Hex1NeuAc1	7.3
Man7	7.2
Man6	6.9
Gn2M3Gn2	6.6
Gn2M3Gn2Hex1NeuAc1	5.5
Gn2M3Gn2Hex1	5.2
Man8	3.4
Gn2M3Gn2F1Hex1	3.0
Gn2M3Gn2F1Hex2NeuAc2	2.8
Gn2M3Gn2Hex2	2.4
Gn2M3Gn2F1Hex2	2.2
Gn2M3Gn1	2.1
Gn2M3Gn1Hex1	2.1
Gn2M3Gn1Hex2NeuAc1	1.4
Gn2M3Hex1	1.3
Gn2M3Gn2NeuAc1	0.1
High Mannose	30.5
Complex (Gn)(Gn)(F)	19.7
Complex longer than GnGnXF including NeuAc	49.8

Gn2M3GnF2XHex	4.5
Gn2M3Gn2F2XHex	4.5
Gn2M3Gn2F2XHex2	4.4
Gn2M3Gn2F3XHex2	4.2
Gn2M3Gn2FXHex2	3.3
Gn2M3GnFXHex2	2.7
Gn2M3Gn2FXHex	2.3
Gn2M3GnF2XHex2	2.0
Man7	2.0
Gn2M3GnFHex2	0.8
Gn2M3GnFHex	0.8
Gn2M3Gn2F	0.7
Gn2M3FXHex	0.5
Gn2M3GnFHex3	0.4
Gn2M3GnF	0.3
High mannose	2.0
Complex (Gn)(Gn)(X)(F)	56.4
Complex longer than GnGnXF including Lewis	41.7

CHO

Glycosite N585 (peptide NFANSSYVQY-M)	
Glycan	Relative Abundance (%)
Gn2M3Gn2F1Hex2NeuAc2	22.4
Gn2M3Gn2F1Hex1NeuAc1	15.5
Gn2M3Gn2F1	9.1
Gn2M3Gn2F1Hex2NeuAc1	8.1
Gn2M3Gn3F1Hex3NeuAc3	6.4
Gn2M3Gn2F1Hex1	5.2
Man5	4.9
Gn2M3Gn2F1Hex2	4.4
Gn2M3Gn2F2Hex3NeuAc1	3.2
Gn2M3Gn3F1Hex3NeuAc2	2.3
Man6	1.9
Man7	1.9
Man8	1.7
Gn2M3Gn1F1	1.3
Gn2M3Gn3F1Hex2NeuAc2	1.3
Gn2M3Gn3F1Hex1NeuAc1	1.1
Gn2M3Gn3F1	1.1
Gn2M3Gn2Hex2NeuAc1	1.0
Gn2M3Gn2	1.0
Gn2M3Gn2Hex2NeuAc2	0.8
Gn2M3	0.8
Gn2M3Gn1	0.7
Gn2M3Gn3F1Hex3NeuAc1	0.7
Gn2M3Hex1	0.7
Man9	0.7
Gn2M3Gn2Hex1	0.5
Gn2M3Gn1Hex1	0.5
Gn2M3Gn1Hex2	0.4
Gn2M3Gn3F1Hex1	0.2
Gn2M3Gn2Hex2	0.1
High Mannose	12.4
Complex (Gn)(Gn)(F)	13.0
Complex longer than GnGnXF including NeuAc	74.6

BY2

Glycosite N585 (peptide NFANSSYVQY-M)	
Glycans	Relative Abundance (%)
Gn2M3Gn2FX	46.9
Gn2M3Gn2F3XHex2	14.0
Gn2M3GnFX	12.5
Gn2M3Gn2F2XHex	10.5
Gn2M3Gn2F2XHex2	6.1
Gn2M3FX	3.2
Gn2M3Gn2FXHex	3.0
Gn2M3GnFXHex	1.7
Man5	1.3
Gn2M3GnF2XHex	0.8
High mannose	1.3
Complex (Gn)(Gn)(X)(F)	62.6
Complex longer than GnGnXF including Lewis	36.1

Supplementary Table 3.

Oxonium	*m/z*
HexNAc	204.0866
NeuAc-H_2O	274.0921
NeuGc-H2O	290.087
NeuAc	292.1027
NeuGc	308.0976
HexHexNAc	366.1395
HexNeuAc	454.1561
HexNAcHexNeuAc	657.2354
HexNAcFucHexNeuAc	803.2928
